# Regulation of transforming growth factor-beta1 by circANKS1B/miR-515-5p affects the metastatic potential and cisplatin resistance in oral squamous cell carcinoma

**DOI:** 10.1080/21655979.2021.2005221

**Published:** 2021-12-14

**Authors:** Jiawei Yan, Hongyan Xu

**Affiliations:** a Chongqing Key Laboratory of Oral Diseases and Biomedical Sciences, Chongqing Municipal Key Laboratory of Oral Biomedical Engineering of Higher Education, Stomatological Hospital of Chongqing Medical University, Chongqing, P.R. China; b Department of Stomatology, Shaanxi Provincial People’s Hospital, Xi’an, P.R. China

**Keywords:** OSCC, circANKS1B/miR-515-5p, TGF-Β1, metastasis, chemoresistance

## Abstract

Oral squamous cell carcinoma (OSCC) is the most common oral cancer, with an increasing worldwide incidence and a worsening prognosis. Emerging evidence confirms that circular RNAs (circRNAs) play a critical role in tumor progression via sponging miRNAs. A previous study substantiated the function of circANKS1B in several cancers. However, its role in OSCC remains unclear. This study revealed the high expression of circANKS1B in OSCC tissues and cells. Moreover, the expression level of circANKS1B was highly positively correlated with the expression of transforming growth factor-beta1 (TGF-β1) in OSCC tissues. Additionally, overexpression of circANKS1B enhanced the protein expression of TGF-β1 in OSCC cells, while its inhibition reduced TGF-β1 protein levels. Noticeably, the loss-function of circANKS1B restrained OSCC cell invasion, migration, and epithelial to mesenchymal transition (EMT) by decreasing N-cadherin expression and enhancing E-cadherin expression. Furthermore, the knockdown of circANKS1B sensitized OSCC cells to cisplatin by suppressing cell viability and increasing cell apoptosis and caspase-3 activity. Mechanically, bioinformation software (circinteractome and starBase 3.0) and dual-luciferase reporter assays corroborated that circANKS1B could sponge miR-515-5p. Moreover, miR-515-5p could directly target TGF-β1 to suppress its expression. Importantly, inhibition of miR-515-5p or supplementation with TGF-β1 overturned the effects of circANKS1B knockdown on cell invasion, migration, and cisplatin resistance. Thus, these findings highlight that circANKS1B might act as an oncogenic gene to facilitate the metastatic potential and cisplatin resistance in OSCC by sponging miR-515-5p to regulate TGF-β1. Collectively, circANKS1B may be a promising target for therapy and overcoming chemoresistance in OSCC.

## Introduction

Oral cancer ranks as a proverbial malignancy with over 377, 000 new cases and 177, 757 deaths worldwide [1]. Oral squamous cell carcinoma (OSCC) is a heterogeneous group of cancers arising from the oral cavity and accounts for about 90% of oral cancers. Over the past few years, epidemiological studies have confirmed the notable increase in incidence and mortality of OSCC around the world, such as in the UK [2]. Patients with advanced OSCC usually experience a high degree of local invasiveness and metastasis. Currently, a multidisciplinary approach combining chemotherapy, radiation therapy, and surgery has yielded satisfactory outcomes in the treatment of early OSCC. However, the high metastatic characteristics and chemoresistance usually limit the application of current curative treatment options and lead to undesired efficacy in patients with OSCC. As a result, the 5-year survival rate among patients with OSCC is still poor, at less than 60% [3,4]. Thus, to better elucidate the underlying mechanism involved in metastasis and chemoresistance acquisition in OSCC, it is essential to develop new therapeutic strategies against OSCC.

Transforming growth factor β (TGF-β, also known as TGF-β1) is a disulfide-linked dimeric protein that is deregulated in various diseases, such as carcinoma. Currently, TGF-β signaling has been extensively investigated. After activation, TGF-β can bind to its receptor (TGF-βR) to evoke multiple target gene expression to impact diverse cellular processes, including cell apoptosis, invasion, epithelial to mesenchymal transition (EMT), and metastasis [5,6]. Accumulating evidence corroborates the abnormal expression of TGF-β in various carcinomas. In OSCC, the high expression of TGF-β is correlated with a high stage and poor prognosis of OSCC patients [7]. Moreover, overexpression of TGF-βfacilitates tumor growth and metastasis [8,9]. Intriguingly, emerging studies have substantiated the key roles of TGF-β in chemoresistance including OSCC [10,11]. Currently, the crucial function of TGF-β has made it a challenging target for cancer prevention and therapy [12].

Circular RNAs (circRNAs) are an emerging new class of endogenous noncoding RNAs that exist in the genome at thousands of gene loci and have a special looped structure with jointed 3ʹ heads and 5ʹ tails. In recent years, many studies have reevaluated their important functions in the progression of various diseases by regulating gene expression. Currently, circRNAs exhibit aberrant expression in multiple types of cancers and have been used as novel cancer biomarkers [13]. Abundant evidence has implicated circRNAs in multiple cancer cell biological processes, including cell growth, invasion, and chemoresistance, via interaction with miRNAs [14,15]. For instance, circPKD2 affects OSCC progression by sponging the miR-204-3p/ adenomatous polyposis coli 2 (APC2) axis [16]. Among them, CircANKS1B (circ_0007294) is a known circRNA originating from exons 5 to 8 of the ANKS1B gene and has drawn more attention because of its key roles in several cancers [17]. In triple-negative breast cancer (TNBC), high expression of circANKS1B is closely associated with lymph node metastasis and may act as an independent risk factor for breast cancer patients [18]. Furthermore, circANKS1B also acts as a sponge of miR-149 to promote colorectal cancer cell migration and invasion [19]. Analogously, circANKS1B can sponge miR-148-3p and miR-152-3p to activate the TGF-β signaling to accelerate cancer metastasis [18]. Nevertheless, the function of circANKS1B in OSCC remains unclear.

In the current research, we hypothesized that circANKS1B might elicit an important role in the metastatic potential and cisplatin resistance in OSCC. Thus, we sought to investigate the roles of circANKS1B in OSCC cell invasion, migration, and cisplatin resistance. Moreover, we investigated the involvement of TGF-β in these processes and found that circANKS1B might act as an oncogenic gene to facilitate the metastatic potential and cisplatin resistance in OSCC by sponging miR-515-5p to regulate TGF-β1. Thus, this study may provide a promising therapeutic target for OSCC.

## Materials and methods

### Clinical specimens and ethics statement

Thirty tumor tissue and paired paratumor samples were enrolled and obtained from OSCC patients (17 males and 13 females, 38‐65 years) diagnosed in the Shaanxi Provincial People’s Hospital from 2017 to 2019. All specimens were surgically resected from patients with OSCC who did not undergo systemic anti-carcinoma therapy. All study protocols were approved by the Research Ethics Committee of the Shaanxi Provincial People’s Hospital and conducted in accordance with the Declaration of Helsinki. The tissues were stored in liquid nitrogen at −80°C until processed. All written informed consent was obtained from each patient prior to enrollment.

### Cell lines and culture

Human OSCC cell lines (CAL27, SCC9, and SCC090) were purchased from the American Type Culture Collection (ATCC; Manassas, VA, USA). Human normal oral keratinocyte cell line, HOK, was obtained from the BeNa Culture Collection (Suzhou, China). Cells (HOK, SCC090, and CAL27) were cultured in Dulbecco’s modified Eagle’s medium (DMEM) medium, and SCC090 cells were incubated in RPMI-1640 medium supplemented with 10% fetal bovine serum (FBS) (Sigma, St. Louis, MO, USA). For cisplatin resistance experiments, cells were stimulated with cisplatin (Sigma) ranging from 2 μM to 50 μM for 24 h. All cells were housed in an incubator (37°C and 5% CO_2_).

### Cell transfection

The oligonucleotide sequences of miR-515-5p mimics, miR-con, miR-515-5p inhibitor (anti-miR-515-5p), inhibitor control (anti-NC), overexpression plasmid of circANKS1B (hsa_circ_0007294), si-circANKS1B and si-control (si-NC) were obtained from GenePharma Biotechnology (Shanghai, China). For transfection, cells were seeded into 6-well plates and treated with the above plasmids using Lipofectamine 2000 (Invitrogen, Carlsbad, CA, USA), as previously described [9]. The final efficacy of circANKS1B and miR-515-5p expression was analyzed using qRT-PCR.

### Total RNA extraction and qRT-PCR assay

Total RNA was extracted from OSCC tissues and cells using the TRIzol reagents (Sigma). Subsequently, the prepared RNA was used as a template to synthesize cDNA using the PrimeScript RT-PCR Kit (Takara, Japan). Then, gene transcripts were performed using a SYBR Premix Ex TaqTM II Kit (Takara) according to a previous report [16]. The specific primer sequences were purchased from Sangon Biotech (Shanghai, China) and used as follows: circANKS1B (sense, 5ʹ-GAAACCGTCACTGGAGAATTATCA-3ʹ; anti-sense, 5ʹ-AAAGCTGCTTCATGAAGTGCAC-3ʹ), miR-515-5p (sense, 5ʹ-CGGGTTCTCCAAAAGAAAGCA-3ʹ; anti-sense, 5ʹ-CAGCCACAAAAGAGCACAAT-3ʹ) and TGF-β1 (sense, 5ʹ-CCTGCCTGTCTGCACTATTC-3ʹ; anti-sense, 5ʹ-TGCCCAAGGTGCTCAATAAA-3ʹ). For normalization, GAPDH or U6 were used as internal references, and the 2^−ΔΔ*C*t^ method was used to quantify the relative gene expression.

### Western blotting assay

Protein expression was performed using a western blotting assay as previously described [8]. RIPA extraction reagent (Beyotime, Shanghai, China) was used to lyse OSCC cells, and the BCA kit (Beyotime) was applied to quantify the extracted protein concentration. After separation by 12% sodium dodecyl sulfate-polyacrylamide gel electrophoresis (SDS-PAGE), proteins were transferred to a polyvinylidene fluoride (PVDF) membrane (Millipore, Billerica, MA, USA). Then, the membranes were blocked with 5% nonfat milk and incubated with primary antibodies against TGF-β (1:1000), E-cadherin (1:2000) and N-cadherin (1:8000) (all from Abcam, Cambridge, MA, USA) at 4°C overnight. After rinsing with TBST three times, horseradish peroxidase-conjugated secondary antibodies were added and incubated for 2 h at room temperature. The immunoblotting signals were developed using chemiluminescence reagent (Beyotime) and normalized using Image J software.

### ELISA assay

After transfection with circANKS1B vector or si-circANKS1B, the contents of TGF-β in supernatants were detected by ELISA assay [20]. All procedures were conducted according to instructions of the commercial ELISA kits (Beyotime). The concentrations in supernatants were calculated in reference to the standard curves.

### Transwell analysis

Cell invasion and migration were analyzed using the Transwell chambers with 8-μm pore filters (Corning, Corning, NY, USA) [16]. Briefly, the transwell inserts were pre-coated with 0.5-mm thickness of Matrigel (BD Biosciences, Bedford, MA, USA) for cell invasion assay, but not for cell migration. Then, cells were adjusted to 1 × 10^4^ cells/mL and seeded into the upper chamber. The culture medium was added to the low chamber of the Transwell System the culture medium. Approximately 48 h later, the invaded or migrated cells at the bottom surface of filter were fixed with 4% paraformaldehyde, and then stained with 0.1% crystal violet solution. An optical microscope was used to count the number of cells by selecting five random microscopic fields. All experiments were conducted in triplicate.

### Cell viability detection by CCK-8

OSCC cell lines (CAL27 and SCC9) were pretreated with si-NC, si-circANKS1B, anti-miR-515-5p or TGF-β, prior to cisplatin exposure. Then, cells were incubated with 10 μl of CCK-8 solution (Nanjing Jiancheng Bioengineering Institute, Nanjing, China) for 4 h. After that, cell viability was evaluated by measuring the absorbance at 450 nm using a spectrophotometer (Bio Rad, Hercules CA, USA).

### Cell apoptosis evaluation by flow cytometry

Following seeding into 6-well plates, cells were stimulated under the indicated conditions. Then, cells were collected, centrifuged and resuspended in binding buffer. For cell apoptosis assay, cells were incubated with10 μl of Annexin V-FITC and 5 μl of PI according to the protocols provided by the Annexin-V-FITC Apoptosis Detection Kit (Beyotime) [16]. After incubation in the dark for 15 min, a FACScan flow cytometer (BD Biosciences, San Jose, CA, USA) was used to detect cell apoptosis by calculating the total percent of early apoptotic (Annexin V^+^ /PI^−^) and late necrotic (Annexin V^+^ /PI^+^) cells.

### Assessment of caspase-3 activity

Cells under various treatments were collected, and a specific substrate of Ac-DEVD-pNA (acetyl-Asp-Glu-Val-Asp-p-nitroanilide was used to determine the activity of caspase-3 according to the previous report [21]. All procedures were conducted according to the instructions of the commercial Caspase-3 Activity Detection Kits (Beyotime). Finally, the absorbance at 405 nm was measured.

### Prediction of circRNA-miRNA-mRNA associations

CircInteractome (http://circinteractome.nia.nih.gov/index.html) was used to predict the correlation between circANKS1B and miR-515-5p. The miR-515-5p/TGF-β correlation was analyzed using Starbase 3.0 (http://starbase.sysu.edu.cn/). The structure of circANKS1B was analyzed using circBase (http://www.circbase.org/).

### Luciferase reporter assay

To elucidate the correlation between circANKS1B, miR-515-5p and TGF-β, the wild-type circANKS1B (circANKS1B-wt), mutated-type (circANKS1B-mut), or wild type (wt) and mutant (mut) 3′-UTR of TGF-β were cloned into a luciferase reporter plasmid pGL3 (Promega Corporation, Madison, WI, USA) according to a previous study [18]. Then, cells were con-transfected with the above plasmids and miR-515-5p mimics or miR-con. Approximately 36 h later, the luciferase activity was assessed according to manufacturer’s instructions using a Dual Luciferase Reporter Assay Kit (Promega).

### RNA Immunoprecipitation (RIP) assay

The relationship between circANKS1B and miR-515-5p was analyzed using the Magna RNA-binding protein immunoprecipitation Kit (Millipore) [18]. Briefly, SCC9 cells were treated with RNA lysis buffer to prepare cell lysates. Then, the RIP buffer containing magnetic beads coated with human Ago2 antibody and IgG antibody (used as control) was then added to lysate for further incubation. After collection of the immunoprecipitated RNA, qRT-PCR was performed to detect the RNA enrichment of circANKS1B and miR-515-5p.

### Statistical analysis

All data were analyzed from at least three independent experiments using SPSS 19.0 software. Results are shown as mean ± SD. Statistical comparisons between two groups were analyzed by the Student’s *t*-test, and ANOVA with post-hoc Student–Newman-–Keuls tests was applied for the analysis of three or more groups. The correlation between the expressions of the two genes was evaluated by Pearson’s correlation coefficient analysis. Statistical significance was defined as P < 0.05.

## Results

### Enhanced expression of circANKS1B and TGF-β1 in OSCC tissues and cells

**Figure 1.**
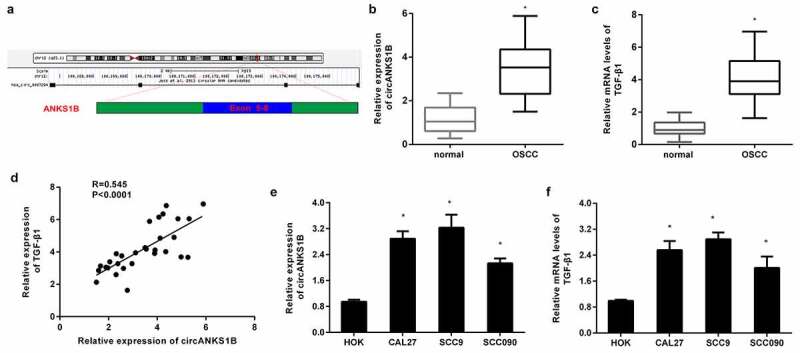
Expression of circANKS1B and TGF-β1 in OSCC tissues and cells. (a) The genomic loci of circANKS1B (has_ circ_0007294). (b, c) qRT-PCR assay was performed to detect the expression of circANKS1B (b) and TGF-β1 (c) in OSCC tissues. (d) Pearson’s correlation analysis was utilized to elucidate the association between circANKS1B and TGF-β1. (e) Expression of circANKS1B in OSCC cells and normal oral keratinocyte HOK cells. *P < 0.05

As shown in Figure 1(a), circANKS1B (Hsa_circ_0007294) is derived from exons 5 to 8 of the *ANKS1B* gene. Of interest, the elevation of circANKS1B expression was validated in OSCC tissues relative to paratumor samples (Figure 1(b)). Furthermore, qRT-PCR also confirmed the higher expression of TGF-β1 in OSCC specimens than that in the paratumor groups (Figure 1(c)). Notably, there was a positive correlation between the expression of circANKS1B and TGF-β1 in OSCC tissues (Figure 1(d)). Likewise, the expression of circANKS1B was increased in OSCC cells in contrast to that in normal oral keratinocyte HOK cells (Figure 1(e)).

### circANKS1B positively regulates TGF-β1 expression in OSCC cell lines

**Figure 2.**
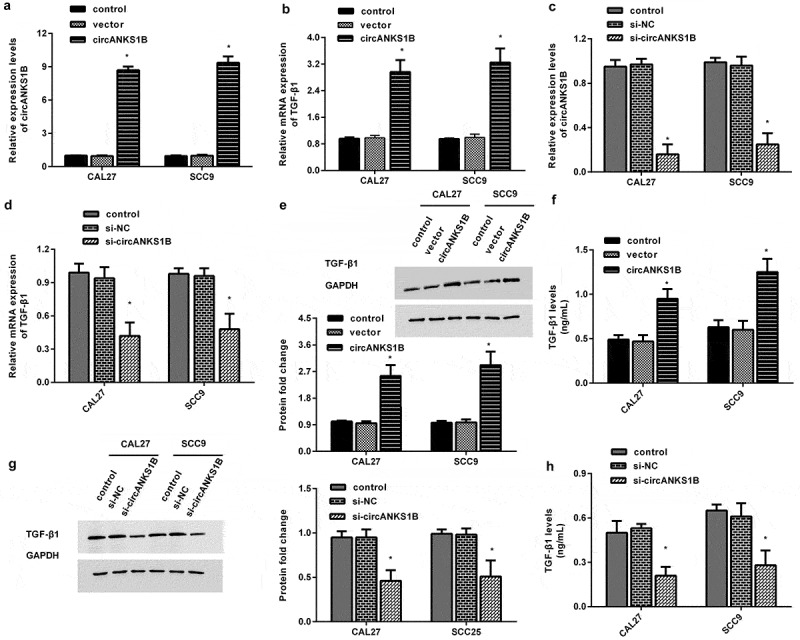
circANKS1B positively regulates TGF-β1 expression in OSCC cell lines. (a) OSCC cells (CAL27 and SCC9 cells) were transfected with the recombinant circANKS1B plasmids. The expression of circANKS1B was then determined. (b) Effects of circANKS1B overexpression on the mRNA levels of TGF-β1. (c) The expression of circANKS1B was detected in cells transfected with si-circANKS1B. (d) Expression of TGF-β1 after si-circANKS1B transfection. (e-h) The expression of TGF-β1 was analyzed by western blotting (e and g) and ELISA (f, h) in cells transfected with circANKS1B vectors or si-circANKS1B. *P < 0.05

TGF-β1 usually acts as an oncogene to accelerate the progression of cancers including OSCC [12]. To corroborate the correlation between circANKS1B and TGF-β1 in OSCC, the OSCC cell lines CAL27 and SCC9 were transfected with circANKS1B plasmids to induce circANKS1B overexpression (Figure 2(a)). Intriguingly, elevation of circANKS1B dramatically enhanced the mRNA levels of TGF-β1 in CAL27 and SCC9 cells (Figure 2(b)). Transfection with si-circANKS1B greatly suppressed its expression in OSCC cells (Figure 2(c)). Importantly, loss of function of circANKS1B decreased the transcript levels of TGF-β1 (Figure 2(d)). Simultaneously, overexpression of circANKS1B enhanced the protein expression of TGF-β1 in OSCC cells, while its inhibition reduced TGF-β1 protein levels (Figure 2(e)).

### Knockdown of circANKS1B suppresses OSCC cell invasion and migration

**Figure 3.**
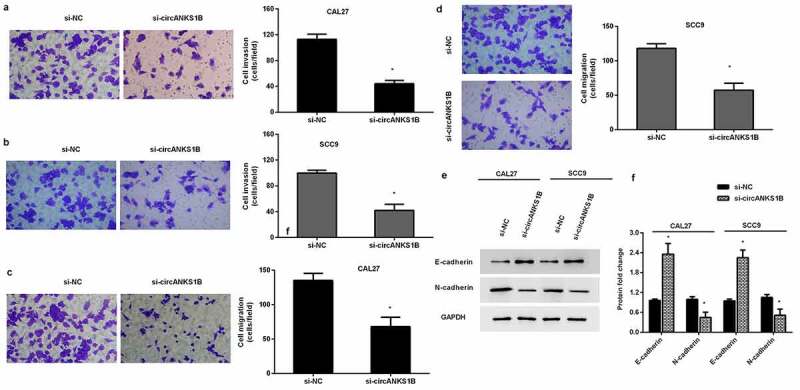
Effects of circANKS1B knockdown on OSCC cell metastatic potential. (a, b) After transfection with si-circANKS1B, cell invasion was evaluated by MTT assay in CAL27 (a) and SCC9 (b) cells. (c, d) Effects of circANKS1B inhibition on cell migration. (e, f) Protein expression of EMT marker E-cadherin and N-cadherin in CAL27 and SCC9 cells. The corresponding bands were quantified by ImageJ software. *P < 0.05

To discern the biological role of cirANKS1B in OSCC progression, we analyzed cell metastatic potential. As shown in Figure 3(a,b), cirANKS1B suppression obviously restrained CAL27 cell (Figure 3(a)) and SCC9 cell invasion (Figure 3(b)). Concomitantly, knockdown of circANKS1B also muted OSCC cell migration (Figure 3(c,d)). Additionally, immunoblotting assay confirmed that si-circANKS1B transfection increased the protein expression of E-cadherin but decreased the protein levels of N-cadherin (Figure 3(e)), indicating the beneficial effects of circANKS1B on EMT in OSCC cells.

### Down-regulation of circANKS1B sensitizes OSCC cells to cisplatin

**Figure 4.**
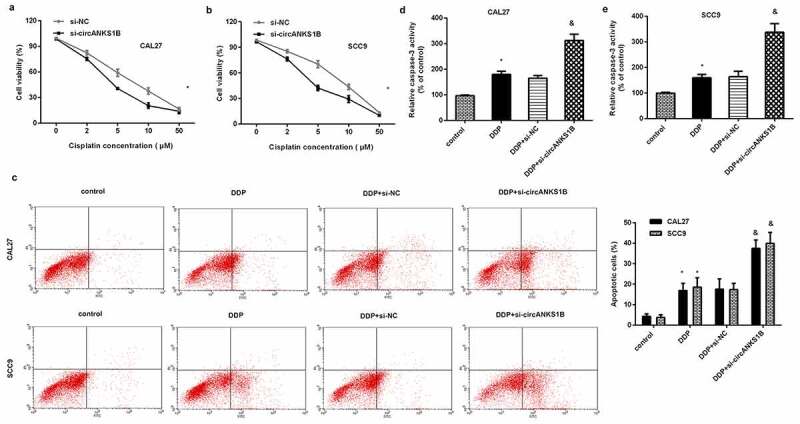
Silencing cicrANKS1B sensitizes OSCC cells to cisplatin. (a, b) After transfection with si-circANKS1B in cells under cisplatin exposure, cell viability was then assessed. (c) The subsequent effects on cell apoptosis were analyzed by Annexin V-FITC/PI staining. (d, e) The activity of caspase-3 was detected. *P < 0.05

We next investigated the function of circANKS1B in OSCC cell resistance to cisplatin. As shown in Figure 4(a,b), cisplatin exposure noticeably inhibited cell viability of CAL27 (Figure 4(a)) and SCC9 (Figure 4(b)) cells. Nevertheless, knockdown of circANKS1B further decreased cell viability under cisplatin condition (Figure 4(a,b)). Additionally, cisplatin treatment-induced OSCC cell apoptosis, which was further aggravated when cells were transfected with si-circANKS1B (Figure 4(c)). Analogously, circANKS1B loss further increased the activity of caspase-3 in OSCC cells relative to the cisplatin-treated groups (Figure 4(d,e)).

### circANKS1B serves as a sponge for miR-515-5p to regulate the expression of TGF-β1

**Figure 5.**
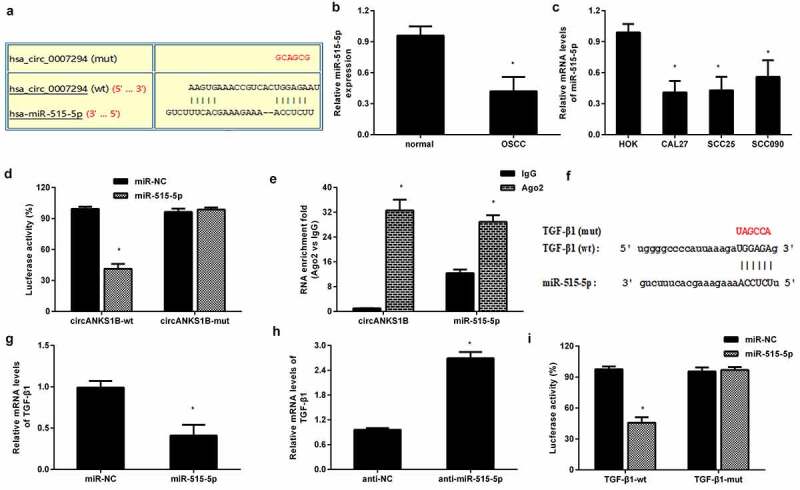
circANKS1B acts as a sponge for miR-515-5p to regulate TGF-β1expression. (a) The potential binding site between circANKS1B (circ_0007294) and miR-515-5p. (b) The expression of miR-515-5p in normal and OSCC tissues. (c) The expression of miR-515-5p in OSCC cells. (d) After co-transfection with circANKS1B-wt/mut luciferase reporter plasmids and miR-515-5p mimics, the luciferase activity was determined. (e) The relationship among circANKS1B and miR-515-5p was determined by RIP assay. (f) Starbase 3.0 was applied to predict the binding site between miR-515-5p and TGF-β1. (g-i) The relationship among miR-515-5p and TGF-β1 was analyzed by qRT-PCR and luciferase activity assay

Emerging evidence substantiates the critical roles of circRNAs in the development of cancers as miRNA ‘sponges’. The bioinformation circinteractome software predicted a potential binding site for miR-515-5p (Figure 5(a)). Noticeably, a recent study has corroborated that miR-515-5p may elicit anti-carcinogenic effects in several carcinomas [22,23]. Importantly, the expression of miR-515-5p was markedly decreased in OSCC tissues (Figure 5(b)) and cells (Figure 5(c)). A Dual-Luciferase Reporter Assay demonstrated that circANKS1B-wt transfection dramatically reduced luciferase activity but not circANKS1B-mut, indicating a direct interaction between circANKS1B and miR-515-5p (Figure 5(d)). Furthermore, RIP assay confirmed the higher levels of circANKS1B and miR-515-5p in anti-Ago2 groups than in the IgG groups (Figure 5(e)). Starbase 3.0 software validated a potential binding site to miR-515-5p in the 3′ UTR of TGF-β1 (Figure 5(f)). QRT-PCR assay confirmed that miR-515-5p overexpression obviously reduced the mRNA levels of TGF-β1 (Figure 5(g)) and its knockdown achieved the reversed efficacy (Figure 5(h)). Additionally, reintroduction of miR-515-5p strikingly decreased the luciferase activity in CAL27 cells transfected with TGF-β1-3′ UTR (wt), implying that TGF-β may act as a direct target of miR-515-5p.

### circANKS1B affects the metastatic potential and cisplatin resistance by sponging miR-515-5p/ TGF-β1

**Figure 6.**
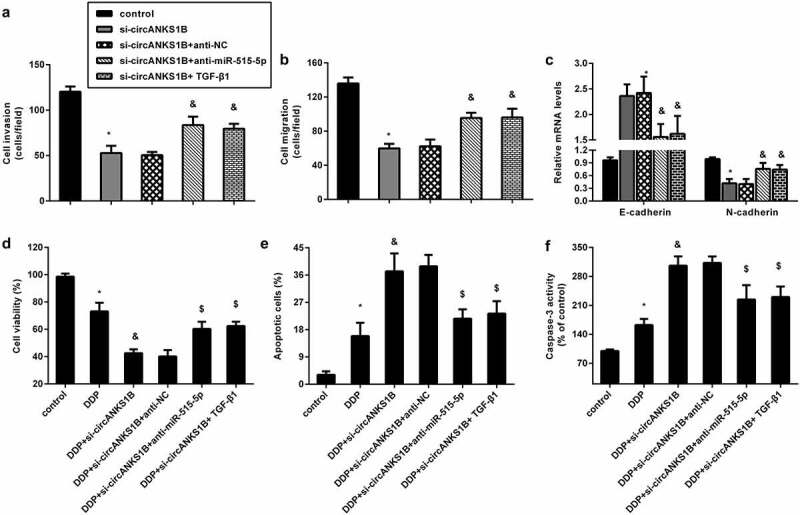
circANKS1B regulates the metastatic potential and cisplatin resistance by sponging miR-515-5p/ TGF-β1. (a-c) CAL27 cells were treated with si-circANKS1B, anti-NC, anti-miR-515-5p or TGF-β1. Then, cell invasion (a), migration (b), and EMT marker expression (E-cadherin and N-cadherin) (c) were analyzed. *P < 0.05 vs. control group, ^&^P < 0.05 vs. si-circANKS1B group. (d-f) Cells under si-circANKS1B, anti-NC, anti-miR-515-5p or TGF-β1 were exposed to cisplatin. Then, cell viability (d), apoptosis (e) and caspase-3 activity (f) were determined. *P < 0.05 vs. control group, ^&^P < 0.05 vs. DDP-treated group, ^$^P < 0.05 vs. DDP and si-circANKS1B-treated group

To further discern the underlying mechanism of circANKS1B in OSCC, we introduced miR-515-5p knockdown and exogenous TGF-β. As shown in Figure 6(a,b), inhibition of circANKS1B strikingly suppressed cell invasion and migration in CAL27 cells, which was reversed by miR-515-5p knockdown or TGF-β1 addition. Similarly, blocking circANKS1B enhanced E-cadherin expression and decreased N-cadherin expression; however, these effects were abrogated after miR-515-5p inhibition or TGF-β1 treatment (Figure 6(c)). Additionally, circANKS1B suppression sensitized cells to cisplatin by decreasing cell viability (Figure 6(d)) and increasing cell apoptosis (Figure 6(e)) and caspase-3 activity (Figure 6(f)). Nevertheless, transfection with anti-miR-515-5p or TGF-β offset the efficacy of circANKS1B knockdown on cisplatin resistance.

## Discussion

OSCC accounts for over 90% of oral cancers and has become a global public health problem due to its high morbidity and mortality. High metastasis and drug resistance limit the efficacy of current therapeutic options for OSCC patients. In the past few years, several studies have highlighted the potential of circRNAs (known non-coding RNAs) as biomarkers for cancer diagnosis and treatment owing to their structural stability [14,15]. In the current study, we first confirmed the high expression of circANKS1B in OSCC tissues and cells. Analogously, previous findings revealed the enhanced expression of circANKS1B, which might be a promising independent marker for patients with breast cancer because its expression was closely associated with lymph node metastasis [18]. Noticeably, the expression of circANKS1B was highly positively correlated with TGF-β1 expression. Thus, these data indicate the potential of circANKS1B in the progression of OSCC.

TGF-β is a common secreted cytokine that plays a key role in controlling tissue repair, inflammation and embryogenic development. Recently, abundant researches have focused on its contribution to carcinogenesis [5]. Aberrant overexpression of TGF-β has been validated in both human and animal tumor samples, including lung cancer, melanoma and OSCC [6]. Intriguingly, the current research highlighted the high expression and positive correlation between TGF-β and circANKS1B in OSCC tissues. In addition, the gain- and loss-of-function of circANKS1B resulted in increased and decreased expression of TGF-β. These findings suggest that circANKS1B may positively regulate TGF-β expression in OSCC. *In vivo* and *in vitro* studies have confirmed that TGF-β enhancement leads to the high proliferation, invasion and metastatic potential of cancer cells including OSCC cells [12,20]. Moreover, TGF-β may act as a promising predictor of tumor recurrence and poor prognosis in patients with OSCC [7]. These data prompted us to investigate the function of circANKS1B in the metastatic potential of OSCC. As expected, knockdown of circANKS1B notably suppressed invasion, migration and EMT of OSCC cells. Moreover, TGF-β supplementation reduced the inhibitory efficacy of circANKS1B suppression on cell invasion, migration and EMT, implying that circANKS1B may facilitate OSCC metastasis by enhancing TGF-β.

Apart from the pro-metastasis potential of TGF-β, a large body of evidence indicates that TGF-βcan also activate target genes to impact chemoresistance in cancer [11]. For instance, TGF-β can bind to its receptor TGF-βR to trigger a complex downstream signaling, leading to chemoresistance in liver cancer [24]. Moreover, targeting TGF-β increases chemosensitivity of cisplatin-resistant OSCC cells [5]. Noticeably, previous studies have mainly focused on the function of circANKS1B in the metastatic potential of breast cancer [18], colorectal cancer [19] and prostate cancer [17]. Based on the correlation between circANKS1B and TGF-β in OSCC, we first investigated the role of circANKS1B in cisplatin resistance. In accordance with our hypothesis, circANKS1B knockdown sensitized OSCC cells to cisplatin, indicating the critical role of circANKS1B in chemoresistance in OSCC therapy.

Recently, increasing evidence has supported the hypothesis that circRNAs, the competitive endogenous RNA (ceRNA), can act as miRNA sponges to regulate multiple pathogenic processes, such as ischemia-reperfusion injury, inflammation and cancer development [14,25,26]. To inspect whether circANKS1B took part in the ceRNA model, we predicted a potential binding site for miR-515-5p using circinteractome software. Importantly, circANKS1B could sponge miR-515-5p, which directly targets TGF-β in OSCC cells. Mounting evidence has illustrated the suppressive function of miR-515-5p in tumor progression [22,23]. For instance, miR-515-5p inhibits breast cancer cell migration and metastasis [23]. Furthermore, miR-515-5p expression is decreased in prostate cancer tissues and can also function as a tumor suppressor in prostate cancer [22]. Of note, overexpression of miR-515-5p or TGF-β both reversed circANKS1B knockdown-mediated inhibition of OSCC cell metastatic potential and cisplatin resistance.

## Conclusions

The current findings highlighted the up-regulation and positive correlation between circANKS1B and TGF-β1 in OSCC tissues. Importantly, circANKS1B suppression elicited the anti-carcinogenic properties in OSCC cell invasion, migration, and cisplatin resistance by sponging miR-515-5p to block TGF-β. Therefore, circANKS1B may act as an oncogenic gene to evoke TGF-β signaling to participate in the development of OSCC. Thus, this study may reveal a promising target for OSCC therapy to overcome chemoresistance.

## Data Availability

All data generated or analyzed during this study are included in this published article.

## References

[1] Sung H, Ferlay J, Siegel RL, et al. Global Cancer Statistics 2020: GLOBOCAN estimates of incidence and mortality worldwide for 36 cancers in 185 countries. CA Cancer J Clin. 2021;71(3):209–249. 33538338 10.3322/caac.21660

[2] Smittenaar CR, Petersen KA, Stewart K, et al. Cancer incidence and mortality projections in the UK until 2035. Br J Cancer. 2016;115(9):1147–1155. 27727232 10.1038/bjc.2016.304 PMC5117795

[3] Ling Z, Cheng B, Tao X. Epithelial-to-mesenchymal transition in oral squamous cell carcinoma: challenges and opportunities. Int J Cancer. 2021;148(7):1548–1561. 33091960 10.1002/ijc.33352

[4] Montero PH, Patel SG. Cancer of the oral cavity. Surg Oncol Clin N Am. 2015;24(3):491–508. 25979396 10.1016/j.soc.2015.03.006 PMC5018209

[5] Chen L, Zhu Q, Lu L, et al. MiR-132 inhibits migration and invasion and increases chemosensitivity of cisplatin-resistant oral squamous cell carcinoma cells via targeting TGF-beta1. Bioengineered. 2020;11(1):91–102. 31906769 10.1080/21655979.2019.1710925 PMC6961592

[6] Fabregat I, Fernando J, Mainez J, et al. TGF-beta signaling in cancer treatment. Curr Pharm Des. 2014;20(17):2934–2947. 23944366 10.2174/13816128113199990591

[7] Lu Z, Ding L, Ding H, et al. Tumor cell-derived TGF-beta at tumor center independently predicts recurrence and poor survival in oral squamous cell carcinoma. J Oral Pathol Med. 2019;48(8):696–704. 31141218 10.1111/jop.12888

[8] Wang BJ, Chi KP, Shen RL, et al. TGFBI promotes tumor growth and is associated with poor prognosis in oral squamous cell carcinoma. J Cancer. 2019;10(20):4902–4912. 31598162 10.7150/jca.29958 PMC6775518

[9] Wang Y, Jia RZ, Diao S, et al. miRNA-101 Targets TGF-betaR1 to retard the progression of oral squamous cell carcinoma. Oncol Res. 2020;28(2):203–212. 31831099 10.3727/096504019X15761480623959 PMC7851522

[10] Brown JA, Yonekubo Y, Hanson N, et al. TGF-beta-induced quiescence mediates chemoresistance of tumor-propagating cells in squamous cell carcinoma. Cell Stem Cell. 2017;21(5):650–64e8. 29100014 10.1016/j.stem.2017.10.001 PMC5778452

[11] Oshimori N, Oristian D, Fuchs E. TGF-beta promotes heterogeneity and drug resistance in squamous cell carcinoma. Cell. 2015;160(5):963–976. 25723170 10.1016/j.cell.2015.01.043 PMC4509607

[12] Colak S, Ten Dijke P. Targeting TGF-beta signaling in cancer. Trends Cancer. 2017;3(1):56–71. 28718426 10.1016/j.trecan.2016.11.008

[13] Zhang HD, Jiang LH, Sun DW, et al. CircRNA: a novel type of biomarker for cancer. Breast Cancer. 2018;25(1):1–7. 28721656 10.1007/s12282-017-0793-9

[14] Patop IL, Kadener S. circRNAs in Cancer. Curr Opin Genet Dev. 2018;48:121–127. 29245064 10.1016/j.gde.2017.11.007 PMC5877416

[15] Verduci L, Strano S, Yarden Y, et al. The circ RNA –micro RNA code: emerging implications for cancer diagnosis and treatment. Mol Oncol. 2019;13(4):669–680. 30719845 10.1002/1878-0261.12468 PMC6441890

[16] Gao L, Zhao C, Li S, et al. circ-PKD2 inhibits carcinogenesis via the miR-204-3p/APC2 axis in oral squamous cell carcinoma. Mol Carcinog. 2019;58(10):1783–1794. 31206208 10.1002/mc.23065

[17] Tao LJ, Pan XY, Wang JW, et al. Circular RNA circANKS1B acts as a sponge for miR-152-3p and promotes prostate cancer progression by upregulating TGF-alpha expression. Prostate. 2021;81(5):271–278. 33556191 10.1002/pros.24102

[18] Zeng K, He B, Yang BB, et al. The pro-metastasis effect of circANKS1B in breast cancer. Mol Cancer. 2018;17(1):160. 30454010 10.1186/s12943-018-0914-x PMC6240936

[19] Li D, Yang R, Yang L, et al. circANKS1B regulates FOXM1 expression and promotes cell migration and invasion by functioning as a sponge of the miR-149 in colorectal cancer. Onco Targets Ther. 2019;12:4065–4073. 31213828 10.2147/OTT.S201310 PMC6536817

[20] Zhang P, Liu Y, Li C, et al. PAPAS promotes oral squamous cell carcinoma by upregulating transforming growth factor-beta1. J Cell Biochem. 2019;120(9):16120–16127. 31099126 10.1002/jcb.28893

[21] Yang X, Zhang Q, Yang X, et al. PACT cessation overcomes ovarian cancer cell chemoresistance to cisplatin by enhancing p53-mediated apoptotic pathway. Biochem Biophys Res Commun. 2019;511(4):719–724. 30827507 10.1016/j.bbrc.2019.02.089

[22] Zhang X, Zhou J, Xue D, et al. MiR-515-5p acts as a tumor suppressor via targeting TRIP13 in prostate cancer. Int J Biol Macromol. 2019;129:227–232. 30685303 10.1016/j.ijbiomac.2019.01.127

[23] Pardo OE, Castellano L, Munro CE, et al. miR-515-5p controls cancer cell migration through MARK4 regulation. EMBO Rep. 2016;17(4):570–584. 26882547 10.15252/embr.201540970 PMC4818771

[24] Bhagyaraj E, Ahuja N, Kumar S, et al. TGF-beta induced chemoresistance in liver cancer is modulated by xenobiotic nuclear receptor PXR. Cell Cycle. 2019;18(24):3589–3602. 31739702 10.1080/15384101.2019.1693120 PMC6927732

[25] Altesha MA, Ni T, Khan A, et al. Circular RNA in cardiovascular disease. J Cell Physiol. 2019;234(5):5588–5600. 30341894 10.1002/jcp.27384

[26] Gao C, Wen Y, Jiang F, et al. Circular RNA circ_0008274 upregulates granulin to promote the progression of hepatocellular carcinoma via sponging microRNA −140-3p. Bioengineered. 2021;12(1):1890–1901. 34002672 10.1080/21655979.2021.1926195 PMC8806606

